# Single and combined effects of parenteral vitamins D_3_, A and K_3_ administration on tibia morphometry, mineral density and ash content in aged Japanese quails

**DOI:** 10.1016/j.psj.2026.106604

**Published:** 2026-02-08

**Authors:** Mahdieh Gholameipour, Heshmatollah Khosravinia, Babak Masouri

**Affiliations:** Dept. of Animal Sciences, Agriculture Faculty, Lorestan University, Lorestan, Iran

**Keywords:** Aged breeder quail, Tibia mineralization, Fat-soluble vitamin, Vitamin D3, Hounsfield unit

## Abstract

A total number of 300 breeder Japanese quails at 40 weeks of age were used to investigate the single and interactive impacts of vitamins D_3_ (0 and 6300 IU), A (0 and 23100 IU) and K_3_ (0, 17 and 34 mg) on tibia mineralization in a 2 × 2 × 3 factorial arrangement in a complete randomized block experiment design. Vitamin preparations were injected subcutaneously (SC) in the back of the neck of birds weekly. Proximal epiphysis and diaphysis diameter of the right tibia bone and its ash percentage were significantly superior in the vitamin D_3_ injected birds by 4.3, 4.9 and 9.3 percent, respectively (*P* < 0.05). Parenteral administration of 6300 IU vitamin D_3_ increased serum level of the same vitamin by 38.5 percent and decreased serum P level (by 12.7 Percent; *P* < 0.05) but not that of calcium (Ca) and albumin (ALB) levels as well as ALP and PTH activity (*P* > 0.05). Robustness index (RI) was significantly greater in the vitamin D_3_ treated birds compared with the corresponding control group (*P* < 0.05). Tibia ash content showed a moderate positive correlation with tibia length (*r* = 0.43; *P* < 0.01) and a negative moderate relation with Robustness index (*P* < 0.01). Serum PTH and ALK activity showed a weak and a moderate correlation coefficient with tibia ash percentage, respectively. No significant interaction was noticed among vitamins concerned in all features assessed (*P* > 0.05). It was concluded that extra nutritional administration of vitamin D_3_ via weekly SC injection, may impose promising effects on bone strength and mineralization in aged laying quails, evidenced by greater tibia ash percentage as well as superior proximal epiphyses and diaphysis diameter.

## Introduction

Optimum dietary levels of the fat-soluble vitamins (A, D, E and K) promote the commercial birds to perform close to their full genetic potential. Vitamin A is crucial for sustaining several physiological functions, including vision, immunity, epithelial cell growth and differentiation, sexual proliferation, and bone health (Shastak et al., 2024). Vitamin D is indispensable for the proper metabolism of calcium (Ca) and phosphorus (P), and the preservation of normal skeletal integrity in animals. It also plays a role in regulation of parathyroid hormone, bone mineralization and mobilization, and it influences the prevalence of bone disorders (Wei et al., 2024). Vitamin K_3_ is well known for its crucial anti-hemorrhagic function in birds since 1936 (Dam and Schonheyder, 1936).

However, the multifaceted impacts of the vitamins A, D and K in many species of the animals, in particular in poultry, has been extensively investigated showing many vital roles beyond the above mentioned their primarily recognized biological significance. Among many conjoint metabolic effects proposed, vitamins A, D, and K are concerned for their single and interactive important roles in bone metabolism and growth (Carmeliet et al., 2015; Essig, 2022; Burns-Whitmore et al., 2024). Vitamin A was shown to promote bone growth and development (Henning et al., 2015; Lemner, 2024; Ling, 2025). Vitamin K is essential for activating proteins involved in bone formation and preventing Ca buildup in soft tissues (Yamaguchi and Weitzmann, 2011; Li et al., 2019; Alonso et al., 2023). Vitamin K involves critical metabolic pathways concerning bone health, preventing vascular calcification (Grimaldi et al., 2025), enhancing brain function (Emekli-Alturfan and Alturfan, 2023; Grimaldi et al., 2025) modulating immune system function, attenuating inflammation, lowering cancer incidence (Sadler et al., 2024). Moreover, it was shown than vitamin K plays a key role in bone mineralization through activation of Osteokalcin (the main vitamin K dependent bone protein) and Gla matrix protein (Galunska et al., 2024). Vitamin K also induces osteogenesis by inhibiting skelerosetin and activation of Wnt/β-catenin pathway (Cui et al., 2021). This vitamin may correspondingly controls osteoclast activity through amelioration of the RANKL/OPG system (Khalaf and Almudhi, 2022). Farm studies also revealed that dietary supplementation vitamin K increased bone strength in broiler chicken (Zhang et al., 2003) and dietary inclusion decreased bone fracture and shell quality in layer hens (Fernandes et., 2009).

Increasing research evidences on the impact of fat-soluble vitamins on bone health and mineralization encourages the poultry industry to welcome studies directing in-depth characterization of the same topic. Bone fragility in laying flocks, particularly those kept in confined battery cages such as quails (Hildebrand et al., 2024; Jansson et al., 2025) and chicken (Szmek et al., 2025), contemplate as a significant welfare and productivity concern. Evidently, fragile bones often lead to enhanced risk of fracture and pain in advanced ages. Bone fragility arises from a combination of factors including genetic predisposition, metabolic stresses engrained basically form the high rate of egg production and the most blamed one as nutritional deficiency, among many others (Jansen et al., 2020; Eusemann et al., 2020).

Obviously fat-soluble vitamins influence bone metabolism in multiple mechanisms. Despite of many reports on age related changes in growth and reproduction in laying commercial birds (Ojedapo, 2013; Nascimento et al., 2014; Taghipour-Shahbandi et al., 2024) research works are infrequent on bone structure parameters in the same birds, in particular at advanced ages. However, in few reports at hand it was shown that bone quality decreases by an increase in porosity, with loss of volume, contributing to age-related osteoporosis in aged laying birds (Yamada et al., 2021). Gan et al. (2020) in a 13-weeks feeding trial using 65-wk-old Hy-Line Brown layers provided diets containing combinations 1- or 2-fold supplantation levels of liquid- or fat-soluble vitamins. They concluded that higher dietary vitamin inclusion levels improved laying performance and egg quality in aged hens. They interestingly attributed the improved parameters to the augmented abundance of beneficial microbiota in the bird’s gut.

In view of the recent concerns on impact of fat-soluble vitamins on different aspect of bone metabolism, investigation on response of bone fragility in aged birds to various combinations of dietary or parenteral administration of these vitamins may provide novel scientific achievements for both researchers as well as poultry industry section. Therefore, the current study aimed to investigate the multifaceted single and interactive impacts of vitamins D_3_, A and K_3_ on tibia mineralization in aged breeder Japanese quails kept in confinement.

## Materials and methods

### Animals, diets, and experimental design

A total of 300 Japanese quails (40 weeks old) with an average initial body weight of 220±10 g was provided from a commercial farm. The birds were allocated in galvanized wire battery cages (× 25 × 35 cm) arranged in 5 rows at a density of 5 birds per cage (4 females and 1 male as an experimental unit) during an 8 weeks experimental period. The study was conducted as a 2 × 2 × 3 factorial arrangement in a complete randomized block design with 12 treatments and 5 replicates (totaling 60 experimental units). The investigated factors included; vitamin D_3_ at two levels (0 and 6,300 IU), vitamin A at two levels (0 and 23,100 IU) and vitamin K_3_ at three levels (0, 17, and 34 mg). The implemented doses supposed to provide total bird’s requirements for a given vitamin for a week, besides the dietary content, based on NRC (1994) recommendations. Vitamin preparations were administrated subcutaneously (SC) in the back of the neck of the birds using a semiautomatic syringe equipment in a weekly schedule. In subsequent weeks the left or right side of the neck was chosen for injection. The birds had free access to water and feed throughout the experimentation period. They were kept in a controlled environment with a constant temperature of 22 ± 1°C, relative humidity of 55 to 60%, a lighting program of 18 hours of light and 6 hours of darkness, and proper ventilation with a minimum of 15 air changes per hour. All experimental procedures were reviewed and approved by the Animal Ethics Committee of Lorestan University.

The basal diet was formulated based on NRC (1994) standards with minor modifications. The composition and the calculated nutrient content of the diet fed are presented in Table 1.

**Table 1:** Composition of the basal diet and calculated chemical composition.

Dietary Ingredients (%)	
Corn	54.81
Soybean meal	32.04
Di calcium phosphate	1.15
Calcium carbonate	6.60
Mineral-vitamin premix^1^	0.10
Salt	0.30
Soybean oil	4.96
DL-methionine	0.04
Chemical Composition (%, calculated)	
Metabolizable energy (kcal/kg)	2900
Crude protein (%)	20
Calcium (%)	2.5
Available phosphorus (%)	0.35
Sodium (%)	0.15
Linoleic acid (%)	2.45
Arginine (%)	1.31
Lysine (%)	1.19
Methionine + Cysteine (%)	0.78
Threonine (%)	0.74
Vitamin A (IU/kg)	81.83
Vitamin D_3_ (IU/kg)	2.3
Vitamin K_3_ (mg/kg)	0.75

^1^ These values per kg of diet include:.

- Vitamins: Vitamin A: 11,000 IU - Cholecalciferol (Vitamin D₃): 2,300 IU - Vitamin E: 121 IU - Vitamin K₃: 2 mg - Vitamin B₁₂: 0.02 mg - Riboflavin: 40 mg - Folic acid: 0.075 mg - d-Biotin: 0.075 mg - Pyridoxine: 4 mg - Ethoxyquin: 0.125 mg.

- Mineral Premix: Manganese: 100 mg - Iron: 80 mg - Zinc: 60 mg - Copper: 8 mg - Iodine: 0.5 mg - Cobalt: 0.2 mg - Selenium: 0.15 mg.

### Blood sample collection and biochemical analysis

At the end of the experimental period, prior to slaughter, approximately 2 mL of blood was collected from the brachial vein of the two randomly chosen female quails from each experimental unit (cage). The blood was placed in EDTA-free tubes for serum biochemistry and kept at ambient temperature (approximately, 27°C) for 2 hours. The samples were then centrifuged at 2000 rpm for 10 minutes to separate the serum from clot (Rifai et al., 2018). The collected serum samples were stored at −20°C after separation. Following thawing, serum parameters including Ca, P (using Pars Azmun kits), parathyroid hormone (PTH) and alkaline phosphatase (ALP) (using Pars Azmun laboratory kits) were determined and an Auto analyzer system (Ciba-Corning Diagnostics Corp., Medfield, MA) (Alagawany et al., 2021). Vitamin D concentration was also assessed using Monobind vitamin D ELISA kit (Monobind Inc., USA) on an Awareness Technology ELISA reader (Awareness Technology Inc., USA). The assay was conducted based on the manufacturer’s instructions and standardized protocols employed in poultry research.

### Bone quality assessment

From each replicate, two female birds were randomly selected and slaughtered at age of 48 weeks. The right tibia bones were collected from the same birds (Zaefarian et al., 2021). Without boiling and after meat removal, the bones were individually packaged in plastic bags and frozen at −20°C pending further analysis.

Prior to analysis, the bones were defrosted for 24 hours under appropriate conditions, and all remaining soft tissues were carefully removed. The length and width (at the proximal and distal epiphyses and mid-diaphysis portion) of each sample were then measured in triplicate and then averaged using an electronic digital caliper (with 0.01 mm precision).

### Bone quality indices calculation

Equation (1) presents the Seedor index, which calculates the ratio of dry bone weight to bone length. This index serves as an indicator of bone density (Evaris et al., 2021):

(1) $$\text{Seedor}\;\text{index}=\frac{\text{Dry}\;\text{bone}\;\text{weight}\;\left(g\right)}{\text{Bone}\;\text{length}\;\left(\text{cm}\right)}$$

Equation (2) defines the Robustness index, representing the ratio of bone length to the cube root of bone weight. This index reflects the mechanical strength of the bone (Evaris et al., 2021):

(2) $$\text{Robustness}\;\text{index}=\frac{\text{Bone}\;\text{length}\;\left(\text{cm}\right)}{\sqrt[{3}]{\text{Bone}\;\text{weight}\;\left(g\right)}}$$

### Bone density assessment

Tibia mineralization was assessed using a Computerized Tomography Scanning system (the Siemens Somatom 2-slice CT scanner, manufactured in Germany, model used at the Faculty of Veterinary Medicine, University of Tehran). For scanning, each bone specimen was positioned in a standardized orientation to minimize measurement error (Anderson et al., 2023a). Prior to scanning, the system was calibrated using a material calibration phantom including water (0 HU), air (−1000 HU), and hydroxyapatite with standard densities (250-1000 mg/cm³). The accuracy of calibration was verified by comparing the measured HU values with reference values (Perilli et al., 2022).

For ash content determination, the bones were first dried at 105°C in an oven for 24 hours (Zhang et al., 2023). The dried samples then ground using a mortar and placed in porcelain crucibles. Subsequently, the samples were ashed in a muffle furnace at 550°C for 24 hours (Wilson et al., 2022) and then cooled in a desiccator prior to weighing. Ash percentage was calculated using Equation (3), representing the mineral content of bone tissue. This index quantifies the proportion of inorganic minerals relative to the organic bone matrix, providing crucial information about bone mineralization status (Anderson et al., 2023b):

(3) $$\text{Ash}\;\text{percentage}\;\left(\%\right)=\left[\frac{\text{Ash}\;\text{weight}\;\left(g\right)}{\;\text{Dry}\;\text{defatted}\;\text{bone}\;\text{weight}\;\left(g\right)}\right]\times 100$$

### Statistical analysis

The data gathered were subjected to analysis of variance with a 2 × 2 × 3 factorial arrangement in a complete randomized block design using mixed model procedure of SAS 9.2 (SAS Institute Inc., Cary, NC). The statistical model (Equation (4)) included the fixed effects of vitamin D_3_ at two levels (0 and 6,300 IU), vitamin A at two levels (0 and 23,100 IU) and vitamin K_3_ at three levels (0, 17, and 34 mg) and their interactions.

(4) Y_ijklm_= µ+BW_i_+D_j_+A_k_+K_l_+(*D* × *A*)_jk_+(*D* × *K*)_jl_+(*A* × *k*)_kl_+(*D* × *A* × *K*)_jkl_+Ԑ_ijklm_

Where; Y_ijklm_ is the analyzed measurement, µ is the overall mean of the population, BW_i_ is the covariate effect of body weight as an independent variable with a continues distribution, D_i_, A_j_ and K_l_ are the independent effects of vitamins D, A and K, respectively, (*D* × *A*)_jk_, (*D* × *K*)_jl_, (*A* × *k*)_kl_, (*D* × *A* × *K*)_jkl_ are possible combinations of the vitamin interactions and Ԑ_ijklm_ is the residual.

Because of a possible influence of variation in initial body weight on the criteria assessed, body weight was included in the analytical model as an independent covariate with normal and continuous distribution for all parameters concerned. Means were separated via Tukey test, and differences between means were considered significant when *P* < 0.05. Pearson’s correlation coefficient was employed to determine any correlation between right tibia bone ash and other traits.

## Results

The mean proximal (upper) epiphysis and diaphysis diameter of the right tibia bone and tibia ash percentage were significantly superior in the vitamin D_3_- injected birds by 4.3 (6.95 vs 6.65 mm), 4.9 (3.18 vs 3.06 mm) and 9.3 (52.59 vs 48.12%) percent, respectively (Table 2; *P* < 0.05). The mean tibia weight and length, distal epiphysis diameter, however, did not differ among the quails receiving parenteral injection of vitamin D_3_ at 0 or 6300 IU (Table 2; *P* > 0.05). No discrepancy in all of the above-mentioned features were observed among the birds receiving either 0 or 23100 for IU vitamin A (Table 2; *P* > 0.05). Parenteral administration of vitamin K_3_ at 17 and 34 mg in a weekly schedule showed no influence on the traits evaluated compared with the corresponding control birds (Table 2; *P* > 0.05). No significant effects were noticed for D_3_ × *A*, D_3_ × K_3_, *A* × K_3_ and *A* × D_3_ × K_3_ interactions on all of the tibial features evaluated (Table 2; *P* > 0.05).

**Table 2:** Effects of parenteral administration of vitamins D_3_, A and K_3_ on tibia ash percentage, tibia weight (g), tibia length (mm), proximal epiphyssis diameter (PED, mm), diaphysis diameter (DD, mm), distal epiphyseal diameter (DED, mm) in older breeder Japanese quail.

Factor / level	Body weight (g)	Tibia ash (%)	Tibia weight (g)	Tibia length (mm)	PED (mm)	DD (mm)	DED (mm)
Vitamin D_3_ (IU)							
0	231.14	48.12^b^	0.82a	54.08^a^	6.66^b^	3.03^b^	5.69a
6300	233.41	52.59^a^	0.88a	53.54^a^	6.95^a^	3.18^a^	5.61a
SEM	2.781	1.241	0.023	0.257	0.071	0.038	0.054
Vitamin A (IU)							
0	236.88	51.25^a^	0.88a	54.26^a^	6.86^a^	3.14^a^	5.64a
23100	227.67	49.46^a^	0.82a	52.68^a^	6.76^a^	3.07^a^	5.66a
SEM	2.588	1.291	0.021	0.341	0.061	0.032	0.042
Vitamin K_3_ (mg)							
0	230.71	47.89^a^	0.85a	54.00^a^	6.78^a^	3.10^a^	5.62a
17	229.83	51.68^a^	0.88a	53.85^a^	6.78^a^	3.11^a^	5.67a
34	236.28	51.49^a^	0.83a	53.68^a^	6.87^a^	3.11^a^	5.66a
SEM	3.157	1.917	0.026	0.413	0.114	0.055	0.051
ANOVA							
D_3_	0.5097	0.0121	0.0836	0.3230	0.0130	0.0147	0.2417
A	0.0095	0.3005	0.0982	0.3240	0.3928	0.2036	0.8580
K_3_	0.2554	0.1365	0.5649	0.3751	0.716	0.9935	0.8213
D_3_ × *A*	0.3976	0.5131	0.8564	0.3212	0.6380	0.9350	0.0000
D_3_ × K_3_	0.9879	0.0915	0.2356	0.3738	0.2327	0.2097	0.3231
*A* × K_3_	0.0024	0.2317	0.6477	0.3763	0.3090	0.6093	0.4992
*A* × D_3_ × K_3_	0.8830	0.9319	0.9004	0.3787	0.1934	0.5436	0.4945

^a,b^ means with no common superscript letter in each column differ significantly (*P* < 0.05).

SEM= Standard Error of Means.

Parenteral administration of 6300 IU vitamin D_3_ increased serum level of the same vitamin by 38.5 percent (109.23 vs 75.96 ng/mL) and decreased serum P level by 12.7 Percent (10.53 vs 12.06 mg/Dl; *P* < 0.05) but not that of Ca and ALB levels as well as alkaline phosphatase (ALP) and parathyroid hormone (PTH) activities (Table 3; *P* > 0.05), compared with the relevant control birds. Serum P and Ca concentrations were declined by 20 (5.55 vs 4.71 mg/dl) and 15.13 (12.55 vs 10.04 mg/dl) percent, respectively, in the birds receiving 23100 IU vitamin A through SC injection in the nape of the neck (Table 3; *P* < 0.05), while serum levels of vitamin D and ALB as well as ALP and PTH activities were remained unchanged (Table 3; *P* > 0.05). No alteration in serum P, Ca, vitamin D and ALB concentration as well as serum ALP and PTH activity were found in the birds treated with 17 and 34 IU vitamin K_3_ than those birds in the pertinent control group (Table 3; *P* > 0.05). Likewise, no change in serum P, Ca, vitamin D and ALB as well serum ALP and PTH activity were detected for D_3_ × *A*, D_3_ × K_3_, *A* × K_3_ and *A* × D_3_ × K_3_ interactive effects (Table 3; *P* > 0.05).

**Table 3:** Effects of parenteral administration of vitamins D_3_, A and K_3_ on serum concentration of Phosphorus (P, mg/dl), Calcium (Ca, mg/dl), vitamin D (Vit. D, ng/mL), Alkaline phosphatase (ALP, U/L), parathyroid hormone (PTH pg/mL), Albumin (ALB; g/dl) in older breeder Japanese quail.

Factor / level	P (mg/dl)	Ca (mg/dl)	Vit. D (ng/mL)	ALP (U/L)	PTH (pg/mL)	ALB (g/dl)
Vitamin D_3_ (IU)						
0	12.06^a^	5.18^a^	75.96^b^	3293.00^a^	3.04^a^	2.47^a^
6300	10.53^b^	5.08^a^	109.23^a^	2969.63^a^	3.67^a^	2.42^a^
SEM	0.612	0.271	6.782	281.801	0.331	0.079
Vitamin A (IU)						
0	12.55^a^	5.55^a^	98.50^a^	2900.66^a^	3.01^a^	2.52^a^
23100	10.04^b^	4.71^b^	86.70^a^	3362.00^a^	3.70^a^	2.38^a^
SEM	0.421	0.261	7.401	274.941	0.301	0.075
Vitamin K_3_ (mg)						
0	10.58^a^	4.70^a^	85.45^a^	3219.50^a^	3.27^a^	2.39^a^
17	11.46^a^	5.26^a^	91.85^a^	3184.50^a^	2.90^a^	2.41^a^
34	11.85^a^	5.42^a^	100.50^a^	2990.00^a^	3.95^a^	2.54^a^
SEM	0.781	0.321	9.011	343.411	0.341	0.075
ANOVA						
D_3_	0.0164	0.7921	0.0010	0.4355	0.1390	0.6872
A	0.0012	0.0307	0.0307	0.2177	0.1096	0.2301
K_3_	0.3916	0.2734	0.4328	0.8867	0.1572	0.5419
D_3_ × *A*	0.2030	0.5052	0.5468	0.3988	0.7363	0.3301
D_3_ × K_3_	0.2384	0.1918	0.2286	0.8230	0.2666	0.9267
*A* × K_3_	0.2847	0.9212	0.6200	0.6615	0.4236	0.7965
*A* × D_3_ × K_3_	0.0852	0.4742	0.1025	0.2930	0.1370	0.8386

^a,b^means with no common superscript letter in each column differ significantly (*P* < 0.05).

SEM= Standard Error of Means.

Seedor index (SI) did not differ in the vitamin D_3_, A or K_3_ injected birds compared with the corresponding control quails (*P* > 0.05). Robustness index (RI) was significantly greater in the vitamin D_3_- treated birds compared with the birds receiving no vitamin D_3_ (Table 4; *P* < 0.05). No difference in HFU for proximal and distal epiphysis and diaphysis, as well as mean HFU was noticed in the aged breeder quails receiving vitamin D_3_, A or K_3_ through a weekly SC injection schedule (Table 4; *P* > 0.05), once more excluding the vitamin D_3_- treated birds which showed superior HFU for proximal tibia epiphysis, than the relevant control birds (Table 4; *P* < 0.05). No discrepancy in HFU measured at proximal epiphysis, diaphysis, distal epiphysis as well as mean HFU was observed for D_3_ × *A*, D_3_ × K_3_, *A* × K_3_ and *A* × D_3_ × K_3_ combined effects (Table 4; *P* > 0.05).

**Table 4:** Effects of parenteral administration of vitamins D_3_, A and K_3_ on Seedor index, Robustness index, Hounsfield unit (HU) for proximal epiphysis (HUP), Hounsfield unit for diaphysis (HUD), Hounsfield unit for distal epiphysis (HUDI) and mean Hounsfield unit (mHU) in aged breeder Japanese quail.

Factor / level	Seedor index	Robustness Index	HUP	HUD	HUDI	mHU
Vitamin D(IU)						
0	0.015^a^	57.86^a^	406.76^b^	809.20^a^	552.23^a^	589.39^a^
6300	0.010^a^	56.30^b^	608.73^a^	1176.80^a^	525.13^a^	692.12^a^
SEM	0.001	0.491	49.712	98.085	84.881	67.071
Vitamin A(IU)						
0	0.011^a^	56.74^a^	506.20^a^	966.60^a^	539.90^a^	592.79^a^
23100	0.013^a^	57.42^a^	509.30^a^	1019.40^a^	537.46^a^	688.72^a^
SEM	0.001	0.531	59.960	237.076	75.876	66.121
Vitamin K3 (mg)						
0	0.012^a^	57.23^a^	502.15^a^	818.15^a^	543.30^a^	621.18^a^
17	0.016^a^	56.34^a^	610.80^a^	1010.05^a^	517.60^a^	712.82^a^
34	0.011^a^	57.68^a^	410.30^a^	1150.80^a^	555.15^a^	588.27^a^
SEM	0.001	0.518	74.071	121.859	101.595	87.498
ANOVA						
D_3_	0.3368	0.0262	0.0265	0.1503	0.8029	0.2909
A	0.3368	0.3188	0.9721	0.8346	0.9821	0.3236
K_3_	0.1245	0.2726	0.1892	0.5594	0.9588	0.5527
D_3_ × *A*	0.6298	0.8304	0.9279	0.4593	0.5572	0.9310
D_3_ × K_3_	0.6648	0.2726	0.2001	0.3159	0.3449	0.6621
*A* × K_3_	0.3354	0.9542	0.5163	0.5779	0.2646	0.3348
*A* × D_3_ × K_3_	0.4711	0.0789	0.224	0.1389	0.4111	0.5642

^a,b^means with no common superscript letter in each column differ significantly (*P* < 0.05).

SEM= Standard Error of Means.

**Fig. 1:**
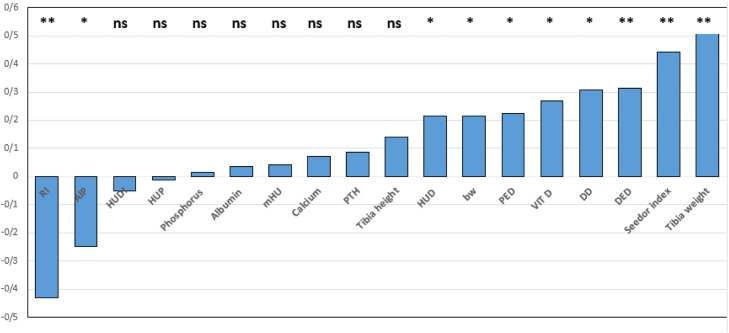
The correlation coefficients of Robustness index (RI), Alkaline phosphatase (ALP), Hounsfield unit for distal epiphysis (HUDI), Hounsfield unit for proximal epiphysis (HUP), Hounsfield unit for diaphysis (HUD), Phosphorus, albumin, mean Hounsfield unit (mHU), calcium, parathyroid hormone (PTH, U/L), HUD, body weight, proximal epiphysis diameter (PED), serum concentration of vitamin D_3_, diaphysis diameter (DD), distal epiphyseal diameter (DED), Seedor index, tibia weight with tibia ash percentage in aged breeder Japanese quail. ns; non-significant,; *P* < 0.05; *P* < 0.01.

The Pearson’s correlation coefficients for tibia ash percentage and all of the evaluated traits were calculated and the results are summarized in Fig. 1. Tibia length showed high positive correlation with tibia ash. Seedor index showed positive moderate correlation (*P* < 0.01) with tibia ash while RI demonstrated a negative moderate association (*P* < 0.01) with the same trait. Serum parameters measured showed weak correlations with tibia ash percentage, with the exception of vitamin D_3_ concentration which found to be moderately correlated (*P* < 0.05) with the same trait. Assessment of HFU at different locations of the right tibia bone showed weak associations with tibia ash content. Finally, serum PTH and ALK activities showed a positive weak (*P* > 0.05) and a negative moderate (*P* > 0.05) correlation with tibia ash percentage, respectively.

## Discussion

In the current study the right tibia bone was chosen as the main indicator to evaluate the impact of the fat-soluble vitamins on skeletal health and mineralization in the aged breeder quails (during weeks 40 to 48 of age). Right tibia bone was considered a decisive evaluating feature in poultry research because its strength and structure are directly interconnected to factors like nutrition (Tompkins et al., 2022a, b), growth rate and leg abnormalities, making it a valuable model for understanding bone health (Lee et al., 2023a). Such importance arises mainly from this fact that the tibia, bears a significant amount of weight, making it sensitive to changes in bone quality (Khan et al., 2023a). Our results showed that tibia weight as well as all tibia morphometric measurements were influenced neither by simple effects of parenteral injection of vitamin D_3_, A and K_3_ nor by their interactive effects with the exception of tibia proximal epiphysis and diaphysis where both significantly found to be greater in those birds receiving 6300 IU vitamin D_3_ (*P* < 0.05).

The lack of influence of vitamin A and K₃ on tibia weight and morphometry agrees with many recently published research findings, such as those of Adhikari et al. (2020), who reported additional vitamin A supplementation to laying hens, regardless of biochemical form, had no effect on either bone mineralization or measures of egg quality. In the contrary, findings of the Stanquevis et al. (2015) study revealed that vitamin K supplementation in quail diet influenced bone density and Ca concentration of the femur and bone density of the tibia. The length of the tibia showed a linear increase according to the levels of vitamin K.

Regarding vitamin K, specifically the K₃ form (menadione), growing evidence from recent studies suggests its role in bone metabolism in poultry may be more complex than previously thought (Ferland, 2022). According to a comprehensive review by Alsaid et al. (2023), although vitamin K is essential for activating gamma-carboxylation-dependent proteins such as osteocalcin (which is vital for bone mineralization), the impact of its supplementation on osteological indicators like tibia weight and dimensions is often influenced by the basal vitamin level in the diet, the presence of other micronutrients, and the bird species. Their study notes that in diets sufficient in vitamin K, additional supplementation may not provide significant benefits to bone morphometric parameters, which aligns with the findings of the current study.

However, the results of the current study did not confirm the findings of Gan et al. (2020) and Huang et al. (2025), where the latter research team concluded that goslings fed 1500 or 2000 IU/kg of vitamin D₃ showed increased tibia ash content (*P* < 0.05). They also found an interaction between vitamins A and D₃ on tibia ash content and bone morphogenetic protein-2 (BMP-2) expression, where at 9000 IU/kg of vitamin A, adding 1000 IU/kg of vitamin D₃ decreased tibia ash (*P* < 0.05). At 1000 IU/kg of vitamin D₃, adding 9000 IU/kg of vitamin A decreased tibia ash (*P* < 0.05). They ultimately concluded that a dietary level of 7000 IU/kg of vitamin A and 2000 IU/kg of vitamin D₃ may be recommended as a combination to optimize feed intake, feed conversion, and tibia mineralization.

It was noticed that vitamin A may positively influence tibia features such as ash content through influencing various cellular functions like proliferation, differentiation, and apoptosis (Yee et al., 2021; Zhang et al., 2022; Khan et al., 2023b). Yee et al. (2021) also concluded that both vitamin A and provitamin A may be potential bone-protecting agents.

Superior certain tibia dimensions in vitamin D_3_ receiving birds were anticipated as almost all previous reports verified that vitamin D_3_ significantly affect bone density and mineralization through increased Ca and P intestinal absorption (Lee et al., 2023a,b) as well as regulations of the other necessary mechanisms involving bone growth (Kianfar et al., 2025).

Our results showed that the vitamin D_3_ effect may realize in a redistribution of mineralization in the proximal and middle parts of the right tibia bone rather than the distal parts. This proposed explanation supported by the increased proximal epiphysis and diaphysis of the bone with no modification in bone weight or distal dimensions. Vitamin D_3_ promotes Ca and P absorption in the intestines and kidneys, supporting bone formation, mineralization, and tibia quality by inhibiting osteoclast-mediated resorption (Zhang et al., 2025). In corroboration of these findings, more recent studies have also emphasized the pivotal role of vitamin D_3_ in bone health. For instance, Wimalawansa (2020) notes that vitamin D3 is essential not only for mineral homeostasis but also for optimal osteoblast function and bone matrix synthesis. Furthermore, research by (Silva et al., 2021) on poultry found that supplementation with 25-hydroxycholecalciferol (25-OH-D3) led to a significant improvement in tibia bone strength indices, including bending strength and bone mineral density, compared to the control group.

It was unexpected that, while serum vitamin D_3_ concentration was increased by almost 40 percent in the quails receiving subcutaneous vitamin D3, no change in the serum level of Ca and P as well as the ALP and PTH activity was noticed in the same birds. In this study, the serum Ca levels were almost low and the corresponding ALB levels were also much higher than those in younger quails from previous studies. These results may be an ordered metabolic response of the liver and endocrine systems in aged hen quails as a consequence of long-term laying metabolic pressure. Aligning with these observations, a study on aging laying hens by de Matos (2022) also showed that the metabolic response to vitamin supplements, including vitamin D_3_, diminishes with age and under prolonged production pressure, and that changes in levels of bone growth factors like IGF-1 may play a role in this. Nevertheless, these findings demonstrate that administration of extra nutritional levels of fat-soluble vitamins to the aged laying quails in either single or combined preparations needs to be further characterized. Such detailed works could result in a recommendable combination of the same vitamins which may be used as an affordable management strategy to improve flock productivity or longevity in the same birds. Since an approximate 40 weeks of long-lasting metabolic pressure has already depleted the main stream of physiological capabilities ordered by genetic predispositions. This perspective is consistent with the recent work of Saito et al. (2023), who emphasize that in aging breeder poultry, focusing on nutritional strategies that aim to improve metabolic efficiency and reduce oxidative stress is far more effective than merely increasing the levels of classical vitamins in the diet.

The effects of the vitamin D_3_ administration on increased mineralization of the proximal portions of the right tibia bone were to some degree supported by the calculated indices as well as the imaging assessment of the bones by Computed Tomography Scan system (CT scan) for evaluating of HFU. The results revealed that the vitamin D_3_-treated birds exhibited superior HFU for proximal epiphysis, compared to the control birds. A greater HFU value in a CT scan generally indicates a more density, and in the case of bone greater mineralization, of the tissue being imaged (Knowles et al., 2016; Buenger et al., 2022). In no report in the literature at hand data on quail bones HFU was found to compare with our finding, however, data from Sung et al. (2025) in a study with broiler chicken, de Silva et al. (2025) in assessment of tibial bone mineral density using two CT-based methods in laying hens and Bahaeddini et al. (2023) on human cases support our findings. The later researchers reported that HFU of the center of the medulla was significantly correlated with the lowest T-score in the proximal (*r* = 0.486, *P* = 0.04) and distal tibia (*r* = 0.458, *P* = = 0.01).

Considering right tibia bone properties as the main indicators to assess overall skeletal health, as most of the previous poultry research, we paid more attention to tibia ash parentage. Evidently, tibia ash content serves as a key metric for judging bone health and efficacy of dietary or parenteral interventions. Gul (2025) reported that a higher tibia ash percentage generally indicates better bone mineralization, the results which extended by Ahmadi et al. (2025) who revealed tibia ash content can be reasonably included in the models reflecting Ca requirements to improve overall skeletal health in quails. In the current study, tibia ash parentage did not differ in the quails receiving parenteral injection of vitamin D_3_, A and K_3_ at the administrated levels, again with the single exception of the vitamin D_3_-treated birds where tibia ash content was significantly greater in those receiving 6300 IU vitamin D_3_. This part of the results was in accordance with the majority of the previous works where scientists mainly confirmed importance of vitamin D_3_ for the maintenance of normal skeletal integrity, bone mineralization and mobilization, and control the incidence of bone disorders in animals (Bikle, 1994; Kebreab et al., 2009).

Considering tibia ash percentage as a major indicator for bone strength and mineralization, the correlation coefficients of the same feature with all the evaluated traits were calculated. Seedor index, distal epiphysis diameter, diaphysis diameter, serum vitamin D_3_ levels and proximal epiphysis diameter were the six traits with superior positive association with tibia ash. In contrast, RI and serum ALP activity were the only two features which exhibited greater moderate negative correlations with tibia ash percentage, respectively. The correlation coefficients calculated for tibia ash with serum related and tibia morphometry traits agree with the figures from the study of Zhang et al. (2019) who revealed tibia growth strongly correlates with body weight. These data may assist the researchers in choosing a set of more relevant features of the tibia bone when investigating the impact of dietary or parenteral intervention of vitamins in bone related poultry research.

Limitations of this study include the small sample size, which may limit generalizability. Additionally, the reliance on computerized tomography for bone density assessment may cause significant cost in poultry research.

## Conclusion

Findings of the current study support this conclusion that among fat soluble vitamins evaluated, only vitamin D_3_ may impose promising effects on bone strength and mineralization in aged breeder quail hens. The main obstacle may be the fact that the bird’s skeleton already depleted form Ca under high metabolic pressure of laying. This conclusion is evidenced by greater tibia ash percent as well as superior proximal epiphysis and diaphysis diameter in the birds receiving parentral administration of vitamin D_3_. In contrary to the many previous studies, our results provide no evidence for interactive effects of vitamins D_3_, A and K_3_ in bone health in aged breeder hen quails based on tibia morphometry and imaging characteristics.

## Funding

This research received no external funding.

## Data and model availability statement

None of the data were deposited in an official repository. The data that support the study findings are available upon reasonable request.

## CRediT authorship contribution statement

**Mahdieh Gholameipour:** Writing – original draft, Visualization, Supervision, Data curation. **Heshmatollah Khosravinia:** Writing – review & editing, Validation, Methodology, Formal analysis, Conceptualization. **Babak Masouri:** Visualization, Project administration, Methodology.

## Disclosures

The authors declare that there is no conflict of interest.
